# Insulin Resistance, Prediabetes, Metabolic Syndrome: What Should Every Pediatrician Know?

**DOI:** 10.4274/jcrpe.2017.S005

**Published:** 2017-12-30

**Authors:** Ahmad Ighbariya, Ram Weiss

**Affiliations:** 1 Ruth Rappaport Children’s Hospital, Clinic of Pediatrics, Haifa, Israel

**Keywords:** Obesity, children, Metabolic syndrome, prediabetes, insulin resistance

## Abstract

The Metabolic syndrome describes a clustering of typical cardiovascular risk factors. The syndrome is also known as “Insulin Resistance syndrome” as a substantial part of the pathophysiology is driven by resistance to the metabolic effects of insulin. The major cause of insulin resistance in childhood is a typical lipid partitioning pattern characterized by increased deposition of lipids within insulin responsive tissues, such as the liver and skeletal muscle and within the viscera. This lipid deposition pattern is also associated with infiltration of intra-abdominal tissues with cells of the immune system, inducing systemic, low-grade inflammation typically observed in insulin resistant obese children and adolescents. Several clues derived from a careful history and physical examination, along with a basic laboratory workup, provide clues in regards to risk stratification in obese children.

## INTRODUCTION

The Metabolic syndrome, also known as Insulin Resistance syndrome or syndrome-X, describes cardiovascular risk factor clustering (CVRFC) in specific individuals (1). The reason for describing these as a syndrome rather than individual and independent risk factors is that they are postulated to be driven by a shared pathophysiological mechanism. The clinical significance of this syndrome is very well established in adults, confirming a significantly increased risk for the development of type 2 diabetes mellitus (T2DM) and coronary heart disease over time (2). While the adult definition of the Metabolic syndrome is well established and can easily be used for clinical purposes, the definition in the pediatric age group is controversial, less stable over time and is harder to utilize clinically (3). There are several reasons for this difficulty, stemming from the normal changes in body proportions in growing children, hormonal effects of normal pubertal development on some of the criteria defining the syndrome and a different balance of the factors governing glucose metabolism between obese children and adolescents compared to adults (4). Some argue that for clinical purposes, the definition of the syndrome should not be utilized and its individual components should be addressed separately (5). This may be true for conveying a clear message to the child and parents, yet the caregiver must understand that there is a common shared mechanism driving the pathophysiology of the development of separate components of the syndrome and that this mechanism should be addressed in order to provide a beneficial clinical outcome. In this review, we first describe the pathophysiology of the syndrome and later provide key clinical insights relevant to the pediatrician.

### Pathophysiology of Insulin Resistance

Reaven (6) was the first to provide a physiological mechanism for the clustering of obesity, dyslipidemia, hypertension and altered glucose metabolism. Reaven (7) suggested that insulin resistance (IR), manifesting as hyperinsulinemia, is the driving factor for the development of dyslipidemia, elevated blood pressure and altered glucose metabolism. As obesity is commonly associated with IR (and is the main cause of IR in childhood), this anthropometric parameter, described using either body mass index (BMI) or waist circumference, serves as part of the syndrome definition. Importantly, there is no uniform definition of insulin sensitivity/resistance. The reason for this is that there is still no standardized assay for measurement of plasma insulin (that must be used to define insulin sensitivity) thus it is difficult to compare results between laboratories using different assays. Moreover, the “gold standard” methodology for measurement of whole body insulin sensitivity is the euglycemic-hyperinsulinemic clamp (8). In this method, a standardized (per body surface area or body weight) insulin infusion is delivered to a fasting patient while in parallel-glucose is infused in order to maintain glucose concentration at a “clamped” fasting level. The steady state glucose infusion rate (in some cases adjusted for ambient insulin concentrations) achieved at the last 30 minutes of the study is defined as the insulin sensitivity of the patient. However, this methodology is used for research purposes only and is not practical for clinical use. Several surrogate indices of whole body insulin sensitivity/resistance have been developed using oral glucose tolerance tests, such as the Matsuda index (9) and fasting samples [such as the homeostatic model for assessment of IR, Homeostatic Model of Assessment-IR (HOMA-IR)] (10). These surrogates have been shown to moderately correlate with “gold standard” measurements in obese, but not necessarily in non-obese, children and adolescents (11), thus their clinical utility is at present not proven. The definition of IR in physiological terms is that greater concentrations of insulin are needed to elicit a physiological effect that was previously induced by lower concentrations of the hormone. Of note, the main factor determining insulin concentrations is its effect on glucose metabolism. Thus, greater plasma glucose, whether derived from endogenous (hepatic glucose production) or exogenous (dietary) sources will result in higher insulin concentrations assuming that beta cell capacity is preserved, which is not the case in patients with diabetes. Insulin sensitivity differs between several insulin-responsive organs so that, for example, in certain conditions hepatic glucose production may be adequately suppressed while muscle glucose uptake may be low on exposure to the same insulin concentration. Moreover, IR in the context of Metabolic syndrome may be present specifically in the insulin signal transduction pathway related to glucose metabolism within a tissue but not in other intracellular elements of this pathway related to other functions such as lipid metabolism or proliferation. For example, this may mean that the resistance to insulin in the suppression of the liver gluconeogenesis pathway could result in higher systemic insulin concentrations yet the response of parallel effects of insulin within the liver [such as very low density lipoprotein (VLDL) synthesis] may not be impaired and thus respond adequately to the higher insulin concentrations by increasing the metabolic flux within that segment of the pathway (12). The main insulin-responsive tissues related to glucose metabolism are the liver, skeletal muscle and adipose tissue. Under fasting conditions, hepatic glucose production is regulated by basal insulin levels while muscle uptake of glucose from the plasma is low and adipose tissue provides free fatty acids (FFAs) via lipolysis as an energy source. In post-prandial conditions, that is when insulin levels are elevated, hepatic glucose production and adipose lipolysis are suppressed while muscle glucose uptake is increased. This is achieved by suppression of gluconeogenesis and glycogen breakdown in the liver and by increased trafficking of the glucose transporter type 4 in muscle. In post prandial conditions, lipogenesis is activated in adipose tissue and lipolysis is suppressed. As indicated earlier, the main regulator of insulin secretion is plasma glucose concentration. If, for example, there is increased IR in skeletal muscle, greater insulin concentrations will be necessary to induce muscle glucose uptake. If hepatic IR is present (i.e., resistance in the insulin signal transduction pathway regulating gluconeogenesis), greater basal insulin concentrations will be necessary to maintain normal fasting glucose levels. Both examples, which usually occur concurrently to some degree, result in relative hyperinsulinemia to which all tissues and organs will be exposed. In this scenario, metabolic pathways regulated by insulin but not necessarily related to glucose will be activated in excess, as there is no resistance in those elements of the insulin signal transduction pathway. For example, in the kidney, insulin stimulates increased sodium reabsorption. In the face of systemic hyperinsulinemia, this will result in excess sodium reabsorption, leading to increased intravascular volume and potentially to elevated blood pressure. It has been shown that insulin resistant individuals have an impaired natriuretic response to increased sodium intake (13), typical of a diet rich in processed food. Similarly, exposure of specific brain nuclei to hyperinsulinemia results in an increased sympathetic discharge, manifesting similarly in elevated blood pressure (14). In the ovaries, theca cells have insulin receptors that respond minimally to normal basal insulin concentrations. However, under conditions of hyperinsulinemia, these receptors induce androgen production resulting in hyperandrogenism (clinically manifesting in hirsutism, oligomennorhea and polycystic ovaries) (15). In the liver, while elevated insulin concentrations may be needed to regulate hepatic glucose production, hepatic, insulin-responsive lipogenesis mechanisms have no resistance and are hyper-activated, resulting in increased VLDL and reduced high density lipoprotein (HDL) particle production, manifesting as increased plasma triglycerides and low HDL-cholesterol concentrations (16,17). Thus, multiple manifestations of the IR syndrome are the result of a normal response of metabolic pathways to increased insulin concentrations that are induced in order to maintain normal glucose metabolism. The reasons for development of IR in insulin responsive tissues are multiple and complex. The common paradigm of this process suggests that accumulation of intracellular lipid (probably via long chain fatty acyl-coenzyme A) induces inhibition of specific components of the insulin signal transduction pathways related to glucose metabolism in liver and muscle (18,19). It is well established that increased intra-myocellular and intra-hepatic lipid are tightly associated with peripheral and hepatic IR respectively (20). Moreover, it has been shown that infusion of intravenous FFAs during a hyperinsulinemic-euglycemic clamp results in an acute reduction of insulin sensitivity (21). Additional factors that may cause acute reductions in liver and muscle insulin sensitivity are an inflammatory stress response, such as that induced by an acute infection or by the use of systemic steroids (22,23,24). In subjects with diabetes, exposure to such stress will result in acute hyperglycemia while in children with normal glucose metabolism these types of stimuli can lead to transient hyperinsulinemia, needed to maintain euglycemia, accompanied by elevated triglycerides. An additional factor linking obesity to increased IR is systemic inflammation (25,26). It is well established that subcutaneous and intra-abdominal lipid depots may be infiltrated by cells of the immune system (mainly macrophages) that have the potential to induce local, as well as systemic, inflammatory activation. Inflammation of hypothalamic nuclei in this scenario may further exacerbate metabolic derangement (27). Similar to fatty acid derivatives within muscle and liver cells, inflammatory cytokines can adversely affect the insulin signal transduction pathway leading to IR. Chronic stress, such as that of chronic disease or emotional stress may have similar effects on systemic insulin responses, resulting in a reduction in whole body insulin sensitivity manifesting as hyperinsulinemia (28). Importantly, the normal physiological hormonal changes of puberty lead to a transient yet substantial reduction in whole body insulin sensitivity during mid-puberty which may resolve by the end of puberty (29,30). Moreover, the impact of sex hormones on components of the Metabolic syndrome may differ between males and females (31). This has relevant implications for the assessment of components of the Metabolic syndrome e, as some may be transiently abnormal in mid-puberty and normalize by the end of puberty. This phenomenon is well established and its significance and impact on the stability of the relevant measurements is a matter of debate (32). Thus, whole body IR manifests clinically in different organs depending on the degree of response to insulin of signal transduction pathways that are not necessarily involved in glucose metabolism. For example, this may manifest as increased activity of the lipoprotein synthetic pathway in the liver (which responds normally to higher systemic insulin concentrations) or in a greater sympathetic discharge. In addition, the insulin resistant, obese child typically shows biochemical evidence of subclinical systemic inflammation.

### Implications of the Pathophysiology of Insulin Resistance on the Clinical Approach to the Obese Child

It is important to identify obese children and adolescents suspected of having an underlying organic cause for their obesity and those who have any obesity related major comorbidities. This group of patients should be referred to a pediatric obesity specialist. In the vast majority of children, the cause of overweight or obesity is a combination of genetic predisposition and environmental factors such as sedentary lifestyle and increased consumption of calorie rich food (33). The proportion of obese children with an organic cause for their weight gain is very low, even within specialist obesity clinics, yet these should be identified and managed appropriately. The primary care physician is responsible for identifying children who are overweight and assessing their vulnerability for developing obesity and its complications (33). This translates into a risk stratification strategy that is aimed at identifying those at greatest risk for present and future obesity related morbidity and focusing on their management.

### Personal Medical History (Table 1)

It is evident that personal medical history details that may raise suspicion of an organic cause of obesity should be sought. Recent accelerated weight gain, substantial weight gain starting in infancy (specifically if accompanied by dysmorphic features), easy bruising, exposure to exogenous corticosteroids, headaches, changes in vision or other clues that raise suspicion of an intracranial lesion, of Cushing’s syndrome or of hypothyroidism should be sought. These are very uncommon causes of obesity and IR, yet should not be missed (33). Starting with pregnancy history, focused questions about maternal gestational diabetes mellitus (GDM), hypertension or any intrauterine growth retardation are crucial for the assessment of the obese child. It is well described that being born small for gestational age (SGA) is associated with a specific pattern of post-natal changes in body composition reminiscent of the lipid partitioning pattern described previously (34). Specifically, those born SGA tend to develop greater intra-abdominal lipid deposition than their appropriate for gestational age counterparts, even prior to the development of obesity, and are at high risk for the presence of the syndrome in adolescence (35,36,37). Moreover, having a head circumference smaller than the 10^th^ centile at birth, indicating significant intrauterine growth retardation, is associated with an increased risk of developing manifestations of IR, and specifically T2DM, in early adulthood (35). Exposure to GDM *in utero* has been shown to affect beta cell function of the developing fetus (38). Obese children who have been exposed to GDM show poorer insulin secretion compared to equally obese children who were not exposed. Being exposed to the hyperglycemic milieu of GDM along with the obligate genetic components associated with T2DM inherited from the parents confer an increased risk of IR in general, and beta cell dysfunction in particular, in the developing fetus which will manifest later in life (39). It is crucial to assess previous anthropometric data using appropriate growth charts and pay attention to the catch-up growth of smaller babies. Accelerated catch-up growth within the first year of life in general and specifically within the first months has been shown to be associated with the lipid partitioning pattern described above which confers greater metabolic risk (40). This is particularly relevant for infants who were born SGA. Infants and older children presenting with significant obesity that developed before the age of five years, and specifically those with significant weight gain in the first year of life, are more suspicious of genetic causes for their obesity. Upon comparing adolescents who were equally obese yet differed in their metabolic phenotype (those with vs. those without the presence of the Metabolic syndrome), it has been shown that early development of obesity as well as length of exposure to obesity were both associated with an adverse metabolic profile (41). While overall caloric intake is very important, specific elements of the diet have been shown to have a greater impact on weight gain and the metabolic profile of the obese child. Specifically, consumption of sweetened beverages has been shown by most studies to be associated with greater risk of obesity in childhood (42). Reduction, and ideally elimination, of sweetened beverages from the diet of an obese child is the basic first step in dietary modification (43). It has been shown that across the globe children may consume a substantial amount of their daily caloric input from such beverages and changing this habit may have a substantial impact on weight and metabolic risk. Fructose consumption has been claimed to be the culprit of lipid deposition in the liver, resulting in increased whole body IR. Short-term substitution of fructose (by eliminating simple sugars in the form of sucrose) with starch, without a change in total calories per day, has recently been shown to reduce intra-hepatic fat and result in significant improvements in glucose and lipid metabolism (44). Thus, specific attention should be paid, when taking the dietary history, to identification of the components of the diet whose change may lead to metabolic improvement even without significant weight reduction. Specific questioning should be devoted to the sleep pattern of the obese child. Firstly, the length of sleep and the ease of awakening should be assessed. Obesity is associated in some cases with an increased tendency to develop obstructive sleep apnea (OSA). OSA has been shown to be associated with further development of IR, probably due to sympathetic activation (45). Thus, children that snore or are suspected of having sleep apnea should undergo a polysomnographic assessment with optional intervention where appropriate. Sleep time should also be assessed as reduced sleep time has been shown to be associated with tendency for obesity (33). Assessment of the physical activity and sedentary behavior of the obese child is mandatory. Greater than 2 hours per day of “media time” (including television, computer and cellphone exposure) have been shown to be associated with a greater risk of obesity. Conversely, any form of physical activity, which does not need to be intense, may have a beneficial impact on body composition and whole body insulin sensitivity. Thus, physical activity may not necessarily lead to weight loss but will have beneficial metabolic effects and should thus be encouraged (46). A thorough history of medication use is also mandatory in obese children. Some medications such as systemic steroids may have a significant adverse impact on weight and on insulin sensitivity (23). Psychotrophic medications, specifically novel anti-psychotics, have been associated with significant weight gain and IR, which is usually observed soon after their initiation (47). A careful family history of children at risk for the presence of Metabolic syndrome is crucial. The syndrome has a strong genetic component as evidenced by twin, as well as parental, studies (48). It is well established that young, lean offspring of parents with type 2 diabetes have greater intramuscular fat and lower whole body insulin sensitivity compared to their counterparts without such family history (49).

### Physical Examination (Table 2)

Accurate anthropometric measurements should be performed in any child and this is equally important in obese children. In addition to plotting standard height, weight and BMI, waist circumference should be measured and, if possible, plotted on appropriate charts. A standard, complete physical examination is mandatory. Specific attention should be given to the presence of dysmorphic features as these, along with weight gain and obesity development in infancy and early childhood, raise more suspicion of genetic obesity syndromes. The presence of acanthosis nigricans (AN) should be sought. The presence of AN, usually seen on the neck and in skin folds, signifies exposure of acanthocytes to hyperinsulinemia (probably interacting with insulin-like growth factor-1 receptors on these cells) (50). Blood pressure should be measured using an appropriately sized cuff. Reference values should be those adjusted for age, sex and height. As some patients suffer from “white coat hypertension”, measurements should be performed in a relaxed and quite atmosphere. If blood pressure levels are elevated on repeated measurements, a 24-hour ambulatory evaluation using a blood pressure halter should be considered. As in any pediatric examination, Tanner staging of pubertal development should be performed, as mid-puberty is characterized by reduced whole body insulin sensitivity. Table 2 shows the main elements to be examined by systems. In addition to these, anthropometric measurements should be recorded (weight, height, waist circumference and calculating BMI).

### Laboratory Workup (Table 3)

The laboratory workup of an obese child suspected of having biochemical manifestations of IR should aim to identify the presence of subclinical clues to this state. A biochemistry panel that includes fasting lipids as well as liver function studies should always be requested. Elevated triglycerides and reduced concentrations of HDL-cholesterol are typical manifestations of IR and are components by definition of the Metabolic syndrome. Moreover, the ratio of triglycerides to HDL-cholesterol is a simple biomarker that identifies increased IR in this age group (51,52). When measured in mg/dL, a ratio greater than 2.25 is a marker of increased IR. Elevated alanine amino transferase (ALT) concentrations, typically less than double the normal range, and without an accompanying elevated aspartate aminotransferase level should raise suspicion of hepatic steatosis (53). However, normal ALT concentrations do not rule out increased hepatic lipid deposition. A urinary sample for the presence of microalbuminuria should be evaluated as early defects in vascular function typically accompany obesity in childhood and are also associated with an adverse metabolic phenotype (54). Thyroid function and an early morning cortisol should be performed as screening for hypothyroidism and Cushing’s, respectively. Other biochemical or hormonal studies should be sent based solely on individual clinical suspicion and should not be performed routinely in all obese patients. The evaluation of glucose metabolism in the obese child is more complicated. Most adult and pediatric definitions of the Metabolic syndrome include a fasting glucose. Fasting glucose is easy to measure, yet impaired fasting glucose (IFG) is only one of the manifestations of altered glucose metabolism in childhood and probably not the major one. Elevated two hour glucose, that is impaired glucose tolerance (IGT), is a stronger predictor of the development of T2DM and of the presence of atherogenic lesions in major arteries in children (55,56). IGT in obese youth is associated with a reduction of first phase insulin secretion, the earliest metabolic lesion that predicts future development of T2DM (57). The evaluation of two hour glucose involves performance of an oral glucose tolerance test. This test may uncover substantial defects in glucose metabolism that might be missed using only a fasting glucose. Importantly, a mid pubertal child may have prediabetes (indicated by either IFG or IGT), which may revert to normal on repeated testing after completion of puberty. This is due to the transient rise in IR during mid-puberty. Albeit being a common phenomenon, this indicates that when being faced with a certain degree of IR, the patient’s beta cell fails to compensate appropriately. This means that while the patient has normal glucose metabolism upon repeated measurements, future decreases in insulin sensitivity (such as physiological ones during pregnancy and normal aging or conditions such as acute diseases, use of corticosteroids and others) may unravel such beta cell defects and manifest as altered glucose metabolism. In an obese child with multiple risk factors, such as a family history of T2DM, AN and/or high waist circumference, an oral glucose tolerance test should be performed as only a fasting glucose my be diagnostically inadequate. Measurement of a fasting insulin level is not recommended by most authorities (34). As the assays for insulin measurement are not standardized, it is difficult to evaluate measurements in comparison to any reference values. Calculation of a HOMA-IR in obese children does not add meaningful clinical information because the correlation between the HOMA-IR and “gold standard” measurements of insulin sensitivity is poor (11). Moreover, slightly elevated insulin concentrations may be present across the entire spectrum of whole body insulin sensitivity in obese children and thus provide no meaningful information that may affect treatment decisions. The only utilization of fasting insulin levels may be for longitudinal follow up of individual patients. Yet the additional information this will potentially add is probably modest and adds little beyond measurement of standard Metabolic syndrome components and standard biochemistry testing.

**Figure 1:**
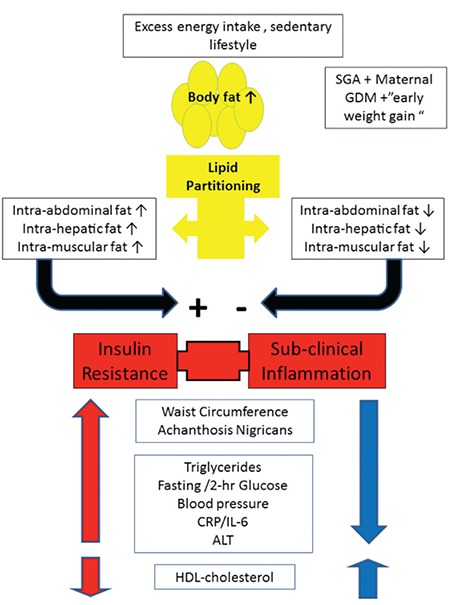
Pathophysiology of insulin resistance and its clinical manifestations. Energy excess and a sedentary lifestyle lead to increased body fat. The ability of subcutaneous fat tissue to expand will determine the lipid partitioning profile. Those with greater ability to expand their subcutaneous depot will have less intra-abdominal and liver/muscle fat deposition and will thus be more insulin sensitive. Those with inability to increase subcutaneous fat will have an unfavorable lipid partitioning profile with increased intra abdominal lipid deposition as evidenced by greater waist circumference and liver/muscle lipid deposition, manifesting as greater insulin resistance and a pro inflammatory profile. This adverse profile leads to elevation of plasma glucose, triglycerides and blood pressure and reduced high density lipoprotein-cholesterol. HDL: high density lipoprotein, CRP/IL-6: C-reactive protein/interleukin-6, ALT: alanine amino transferase, SGA: small for gestational age, GDM: gestational diabetes mellitus

Novel biomarkers, such as adiponectin levels, may provide strong evidence for the presence of IR and altered lipid partitioning (58,59). These molecules are not yet used for standard clinical care yet provide valuable insights into the metabolic phenotype of the obese child. Figure 1 relates the pathophysiology associated with obesity to the parameters of the Metabolic syndrome and summarizes the relationships between them.

### How Do We Interpret the Results of Our Workup?

After investigation of relevant anthropometric and biochemical markers, a risk stratification evaluation of the obese child can be performed. Using the most relevant Metabolic syndrome definition available and relevant to the patient, the presence of risk factors can be quantified. The most appropriate simple definition of the Metabolic syndrome is the International Diabetes Federation definition which provides thresholds for defining the presence of each factor (60). This definition is appropriate for children older than 10 years of age. Most definitions indicate that the presence of a specific number of risk factors, such as obesity and dyslipidemia for example, establishes the diagnosis of Metabolic syndrome. This diagnosis is important as it indicates future health risks for the individual patient. However, care should be exercised as each component of such definitions should not be used as a dichotomous factor. Rather, the metabolic risk conferred by each factor is continuous and does not have a clear threshold effect. It is well established that beta cell function and peripheral IR are associated with elevated two hour glucose levels, even within the normal glucose tolerance range, without meeting the criteria for IGT (61). Thus, for example, a child with a fasting glucose of 95 mg/dL and a two hour glucose of 135 mg/dL does not meet the criteria for IFG or IGT yet may have substantial impairment of beta cell function (61). Similarly, the degree of obesity is important but the lipid partitioning profile is a stronger determinant of the metabolic profile. Thus, an overweight child on the 93^rd^ centile for BMI, who has an increased waist circumference which is a surrogate of increased intra-abdominal lipid deposition may have a much worse metabolic profile compared to a child with a BMI on the 95^th^ centile with low levels of abdominal fat. Similarly, elevated triglyceride concentration, even below the typical threshold of 150 mg/dL may be associated with significant IR and future metabolic risk (62). Thus, individual factors comprising the clinical definitions of Metabolic syndrome should be used as continuous variables as they are associated with a continuous risk. Each component of the syndrome should be assessed longitudinally for its dynamics in response to any intervention. Each obese child should be evaluated for the presence of individual risk factors and those with more metabolic and anamnestic elements are probably those who should be referred for more intensive interventions. Diagnosis of the Metabolic syndrome in an adult is a clear and established indication of increased cardiovascular risk. Diagnosing the presence of the Metabolic syndrome in a child, regardless of the definition used, should be interpreted with caution. It should indicate to the caregiver that multiple metabolic derangements share a common pathophysiology. This translates from a clinical point of view into avoiding trying to address individual components separately. Rather-improving insulin sensitivity, by means of weight loss, dietary modifications, exercise and/or by pharmacological means, will result in improvement of several factors associated with the Metabolic syndrome in parallel. Moreover, meeting the criteria of the metabolic syndrom implies that the child has CVRFC and is at a greater risk for the development of T2DM and cardiovascular disease at an early age. However, failing to meet these criteria is by no means an indication of “healthy obesity”. Rather, it is unclear at present if every element of such definitions has an equal risk implication for the future of the child. Some argue that the obesity component has greater importance while others argue that the presence of IGT has greater impact than other factors. Thus the relative importance of each individual component of the Metabolic syndrome is debatable.

## CONCLUSION

Overweight and obese children and adolescents should be evaluated, keeping in mind that the metabolic morbidity associated with obesity is not necessarily due to the degree of obesity per se. Rather, an in depth evaluation into lipid partitioning and a clinical understanding that the presence of cardiovascular risk factors stems from a shared pathophysiology should guide the caregiver in assessing the metabolic risk and tailoring appropriate intervention for each specific child. Figure 1 depicts the pathophysiology and the physical/laboratory manifestations of the IR syndrome in children.

## Figures and Tables

**Table 1:**
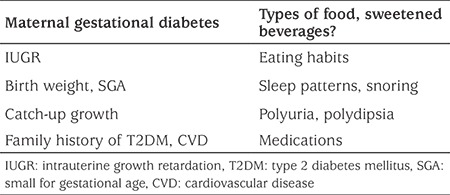
Clues in the history for metabolic syndrome

**Table 2:**
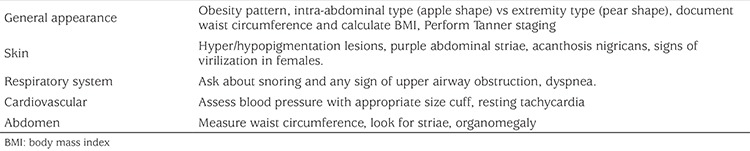
Main elements in the physical examination of the obese child evaluated for the presence of cardiovascular disease risk factors

**Table 3:**
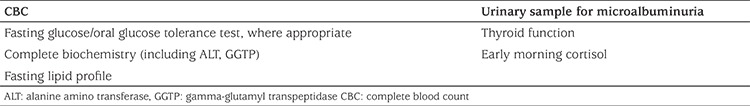
Laboratory tests for evaluating obese children
